# Supplementary material for: Hidradenitis Suppurativa and Comorbid Disorder Biomarkers, Druggable Genes, New Drugs and Drug Repurposing—A Molecular Meta-Analysis
Source: Pharmaceutics. 2021 Dec 26;14(1):44. doi: 10.3390/pharmaceutics14010044 (PMC8779519; doi:10.3390/pharmaceutics14010044)
Supplement: Supplementary file 1 [file pharmaceutics-14-00044-s001.zip › pharmaceutics-1508386-supplementary.pdf]

pharmaceutics

Supplementary Materials: Hidradenitis Suppurativa and Comorbid Disorder Biomarkers, Druggable Genes, New Drugs and Drug Repurposing—A Molecular Meta-Analysis

Viktor A. Zouboulis, Konstantin C. Zouboulis and Christos C. Zouboulis

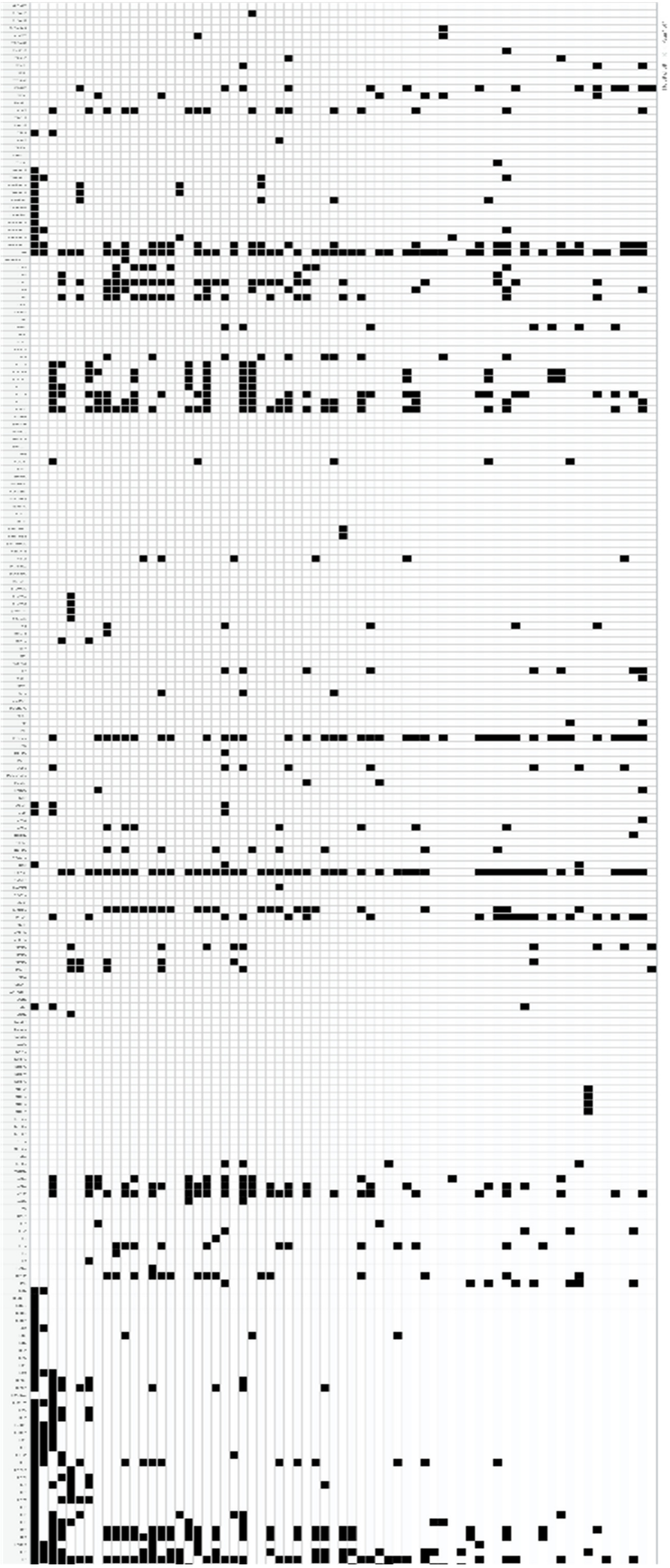

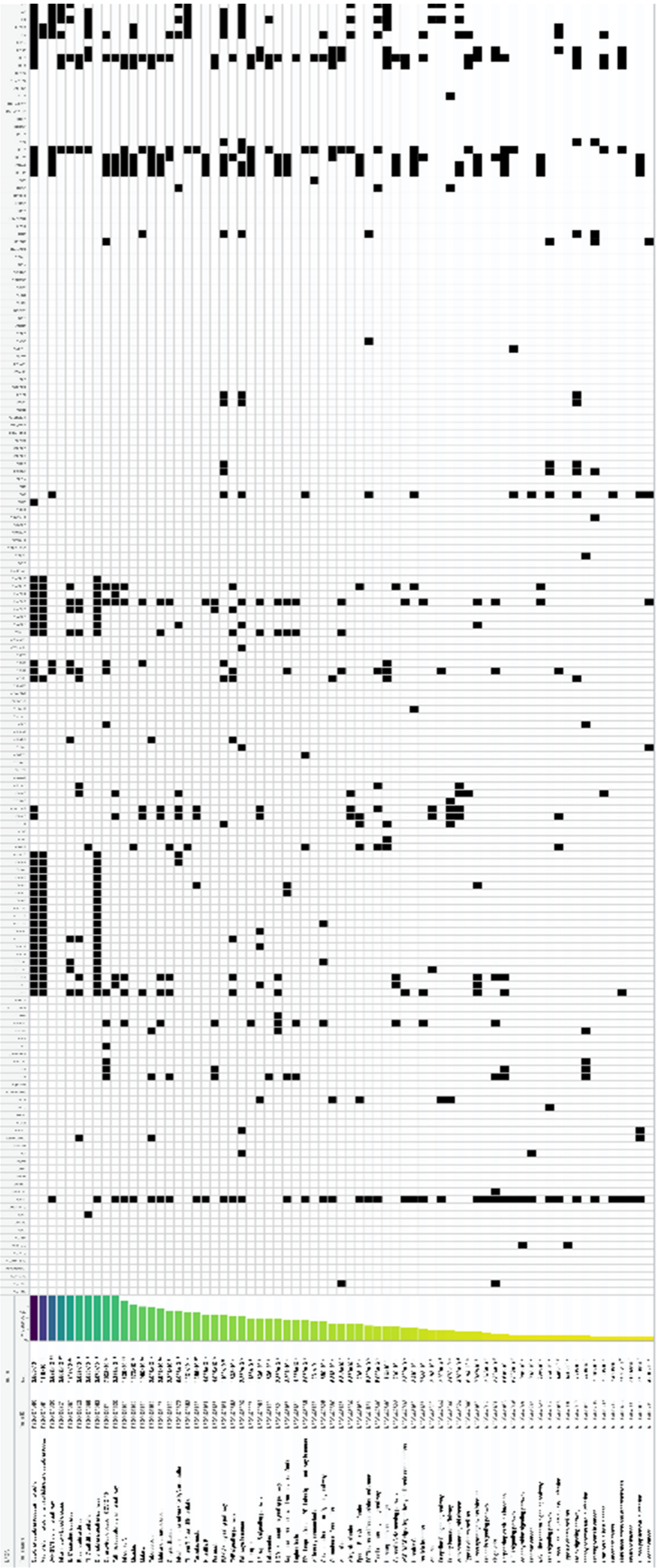

Figure S1. Enrichment of HS-associated DEGs (n = 386) resulting from the comparison of transcriptomic profiles and protein expression studies between lesional HS and non-lesional skin biopsies and blood samples from HS patients and healthy controls, respectively, in signaling pathways.

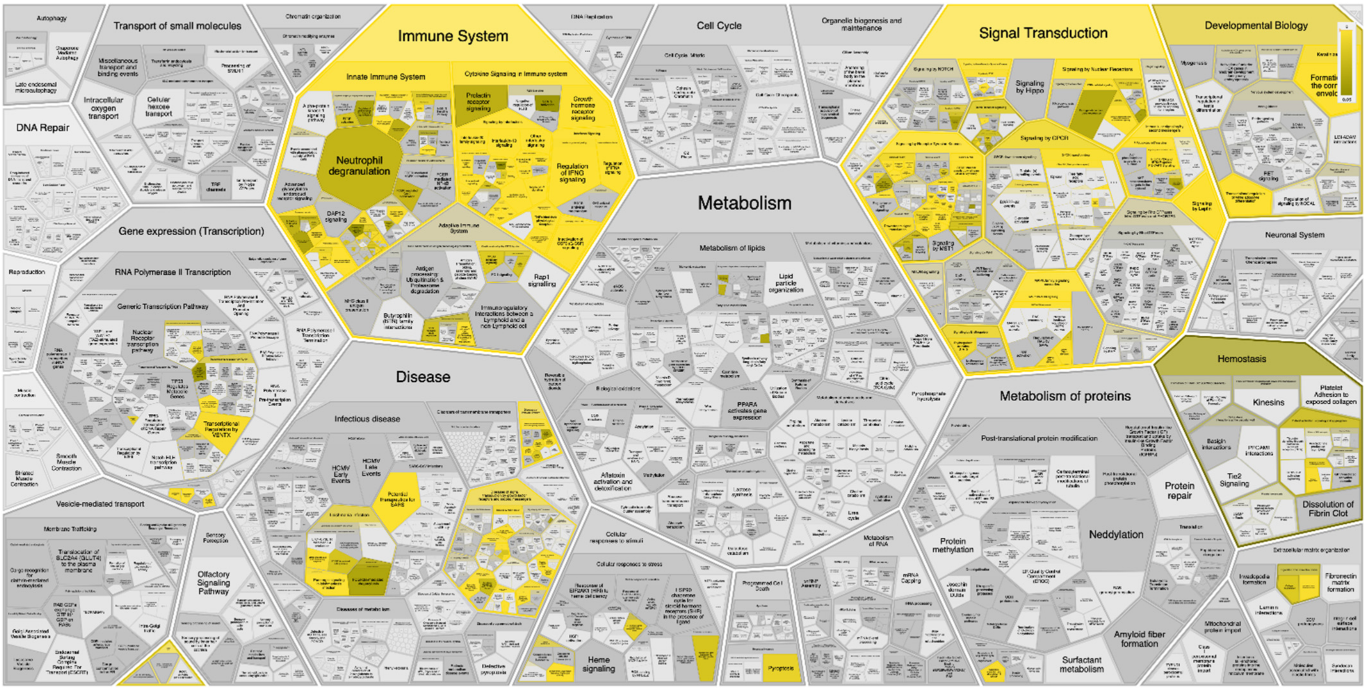

Figure S2: Global REAC evaluation of possibly involved signaling pathways in HS. The intensity of yellow areas and letter size indicate the importance of the specific pathways in HS.

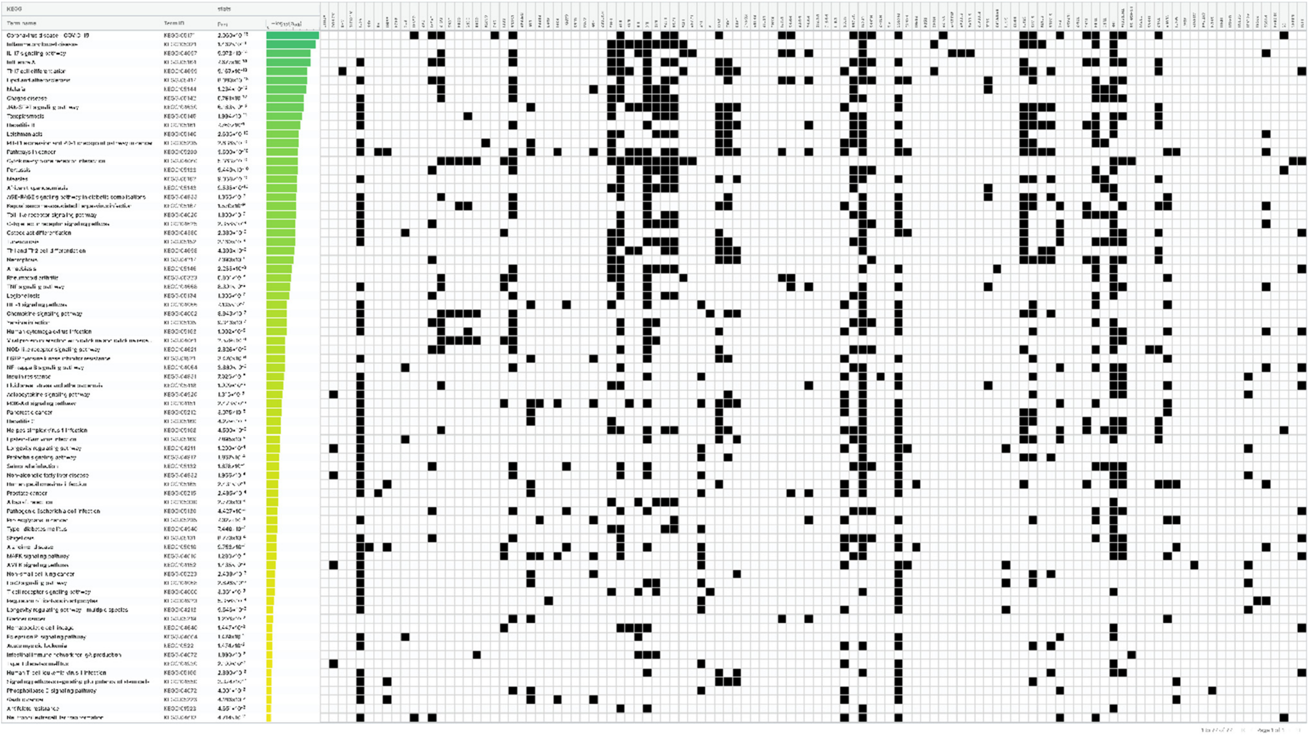

Figure S3. Enrichment of druggable HS-associated genes (n = 116) resulting from the comparison of transcriptomic profiles and protein expression studies between lesional HS and non-lesional skin biopsies and blood samples from HS patients and healthy controls, respectively, in signaling pathways.

Table S1. DEGs resulting from the comparison of transcriptomic profiles and protein expression studies between lesional HS and non-lesional skin biopsies and blood samples from HS patients and healthy controls, respectively. Bold letters indicate druggable genes. Background: white = result reported in a single study; grey = result reported in at least two independent studies in one target (biological material); orange = result reported in two targets; yellow = result reported in three targets. + = upregulation; - = downregulation; +/- = diversified dysregulation in different biological materials; () = lower level of evidence.

	Blood		Skin		
**Gene**	**mRNA**	**Protein**	**mRNA**	**Protein**	**Gene/protein regulation**
ACKR2			[26]		-
ACTN4			[3]		+
ADAM12			[3,27]		+
ADAMDEC1			[27]		+
ADAMTS12			[26]		-
**ADH1C**			[3]		-
**ADIPOQ**		[28]	[27]		-
ADIRF			[3]		-
AGO1			[29]		-
AGO2			[29]		-
**AHR**			[30]		+
**AKR1B10**			[27]		+
**AKT1**			[30]		+
AKT1S1			[30]		+
**ALOX5**			[31]		+
**ALOX5AP**			[31]		+
**ALOX12**			[31]		-
**ALOX15**			[31]		-
ALOXE3			[31]		-
APELA			[27]		+
APOE			[26]		-
**APP**			[26]		+
AQP5			[32]		-
**AR**			[3,33,34]	[35]	+
AS3MT			[3]		-
ATP6V0A4			[27]		-
**AXIN1**				[36]	+
BCL6			[30]		+
**BRD4**			[30]		+
BTC			[3,27,33,34]		+/(-)
**BTK**			[30]		+
C10orf99			[3]		+
C1QTNF6			[3]		+
C3		[27]	[30]		-/+
**C5**		[37]			+
**C5AR1**			[3,30]		+
C5orf46			[33]		-
C7			[27]		-
**CA6**			[27]		-
CAMP			[30]		+
**CASP1**				[38,39]	+
CASP5				[38]	+
CCDC88B			[3]		+
CCDC9			[3]		-
**CCL2**			[40]		+

CCL4				[41]	+
CCL5			[30]		+
CCL11			[42]		+
CCL17			[30]		+
CCL18			[27,30]	[43]	+
CCL19				[26]	+
CCL20			[30,44]		+
CCL21				[26]	+
CCL22			[30]		-
CCL26		[41]	[30]		+
CCL27			[30,33,44]		-/(+)
**CCR2**			[30]		+
**CCR4**	[45]		[30,45]		+
**CCR5**			[30]		+
CCR6			[30]		+
CCR7			[30]		+
**CCR9**			[30]		+
CCR10			[30]		+
CD3G				[46]	+
CD5				[36]	+
CD6				[36]	+
CD19			[45]		+
CD40			[30]		+
CD40LG			[30]		+
CD79a			[45]		+
CD80			[30,38]		+
CD163				[43]	+
CD177			[45]		+
CD244				[36]	+
**CD274**			[30]		+
CDH1				[47]	-
CEBPB			[3]		+
CERS2			[48]		-
**CFD**			[3]		-
CHI3L1		[49]		[50]	+
CLDN8			[27]		-
CLEC3B			[3]		-
COX6B2			[3]		-
CPNE5			[3]		+
**CSF1**			[3,33,34,40]		+
CSF2			[26]		+
CSF3			[51]		+
CST5				[36]	+
CTLA-4			[30]		+
CTNNA1			[26]		-
CTNND1				[47]	-
CXCL1			[27,40,42,44,45]	[40]	+
CXCR4			[3]		+
CXCR5			[30]	[26]	+
CXCL6			[42]		+
**CXCL8**			[30,42,44]	[41]	+
CXCL9			[30]		+
CXCL10		[41]	[30]	[41]	+/(-)

CXCL13		[30,42,45]	[26]	+
CYP2W1		[3]		-
DCD		[27,32,33]	[32]	-
DEFB1		[44,46]		-
DEFB4A		[3,27,30,32,39,44,45,46]	[3,53]	+/(-)
**DEFB4B**	[54]			+
DEFB103B		[46,52]		+
DGCR8		[55]		-
DROSHA		[55]		-
DSC2		[27]		+
EBI3		[40]		+
**EGF**		[3,33,34]		+
EHF		[3]		+
EPGN		[3,33,34]		+
**ERBB4**		[27,32]		-
EREG		[3,33,34]		+
**FABP4**		[27]		-
FABP5		[3]		+
FABP7		[27]		-
FAM118A		[3]		-
FAM74A4		[3]		-
FBN2		[27]		+
**FGF1**		[11]		+
FGF7		[11]		+
FGFBP2		[27]		-
FLG		[30]		+
FOXA1		[32]		-
FOXQ1		[32]		-
FUT6		[3]		-
**GAPDH**	[27]			+
GAS6		[3,33,34]		+
GBP6		[3]		-
GDNF		3,33,34]	[36]	+
GFI1		[26]		-
GFOD1		[3]		-
GHRL	[28]			-
**GJB2**		[3]	[3]	+
**GPD1**		[3]		-
GPR155		[3]		-
GPR68		[3]		+
GSN		[3]		-
GZMH		[30]		+
HAVCR2		[30]		+
HBEGF		[3,33,34]		+
**HGF**		[3,33,34]		+
HIF3A		[27]		-
**HMGCS2**		[27]		-
HRG		[3,33,34]		+
HSCB		[3]		-
HYAL4		[3]		-
ICOS		[30]		+
IDO1		[30]		+
IFNA1		[3,26,30,33, 34]		+

IFNA2		[26]		+
IFNB1		[30]		+
**IFNG**		[3,26,30,33,34,40,44,45,46]		+
IGF2		[3,33,34]		+
IGH			[46]	+
IGHD		[27,30]		+
IGHG3		[27,30]		+
IGK			[45]	+
IGKC		[27]		+
IGKV1D-13		[27,30]		+
IGKV1ORY-1		[33]		+
IGKV3-20		[33]		+
IGL			[45]	+
IGLL1		[33]		+
IGLL3P		[33]		+
IGLV		[27,30]		+
IGLV3-27		[45]		+
IKZF2		[26]		-
IL1A		[3,26,30,33,34,40]	[39]	+
**IL1B**		[26,30,38,40,42,46]	[38,56]	+
**IL1R1**		[30]		+
IL1RN		[26]		-
IL2		[26,30]		+
IL2RA	[49,56]	[30]		+
IL4		[3,30,33,34,40]		+
**IL4R**		[30]		+
**IL5**		[30]		+
**IL6**	[40]	[3,26,30,33,34,40,42]	[40,58]	+
**IL10**		[30,38,44,46]	[52,56]	+
IL10RA		[26]		-
**IL12A**		[59]	[41]	+
**IL12B**		[30]	[36]	+
IL13		[3,30,45]		+/(-)
IL15		[30]		+
IL16		[30]	[41]	+
**IL17A**	[59]	[3,30,33,34,38,39,40,42,44,46,60]	[4,36,38,39,41]	+
IL17C		[45]		-
IL17D		[32]		-
**IL17F**		[30,39,40,42,45]		+
**IL17R**		[3]	[4]	+
IL18		[26,30]	[38]	+/(-)
IL18R1			[36]	+
IL19		[3,30,40]		+
IL20		[30,46]	[46]	+/(-)
IL20RA			[46]	-
IL20RB		[30]		+
IL21		[30,39]		+
IL22		[3,30,40,42,46]	[46]	+
IL22RA1		[30]	[46]	-
IL22RA2			[46]	+
IL23A		[30,40,61]		+
IL23R		[30]		+

IL24		[30,42,46]		+
IL26		[42,46]		+
IL27		[40]		+
IL31		[30]		+
IL32		[30,40,61]		+
IL33		[30]		+
IL34		[42]		-
IL36A	[62]	[30,40,42,45,61]	[39,61]	+
IL36B	[62]		[61]	+
IL36G	[62]	[30,40,42,45]	[61]	+
**IL36RN**			[61]	+
IL37		[32,33,42]		-
**INS**	[63]			+
IRAK1		[30]		+
IRAK2		[30]		+
IRF4		[30]		+
IRF5		[30]		+
IRF7		[26]		+
IRF8		[30]		+
IRF9		[44]		+
IRS1		[30]		+
**ITK**		[30]		+
ITPKA		[30]		+
IVL		[30]		+
JAG2		[26]		-
**JAK1**		[30]		+
**JAK2**		[30]		-
**JAK3**		[3,30]		+
**KCNN2**		[48]		-
KITLG		[11]		+
KLK6		[30]		+
KMT2D		[26]		-
KRT2		[33]		-
**KRT6A**		[3,32]	[3]	+
KRT6B		[3]		+
KRT6C		[3]		+
KRT14		[3]		+
**KRT16**		[3,27,30,32]	[3]	+
KRT19		[32]		-
KRT31		[32]		-
KRT73		[32]		-
KRT74		[32]		-
KRT77		[27,32,33]	[32]	-
KRT79		[32]		-
**KYNU**		[30]		+
LAG3		[30]		+
LCE2B		[33]		-
LCE3D		[32]	[32]	+
LCE5A		[33]		-
LCN2	[64]			+
LEP	[28]			+
LGR5		[27,32]		-
LORICRIN		[30]		+

**LTA4H**		[27,65]	[31]		-/(+)
MAZ			[3]		+
**MMP1**			[3,40]	[3]	+
**MMP3**			[40]	[40]	+
**MMP8**				[66]	+
**MMP9**			[3,30,40]	[3]	+
**MMP10**			[40]		+
**MMP12**			[27,30]		+
**MST1**			[3]		+
**MTOR**			[30]		+
**MYD88**			[26]		+
MZB1			[33]		+
NAA40			[31]		-
NAMPT		[28,63]			+
NCSTN	[67]				-
**NFKB1**			[26]		+
NGF			[3,33,34]	[36]	+
NKX2-3			[26]		-
**NLRP3**				[38]	+
NR1D1			[32]		-
**NR1H2**			[26]		-
OAS2			[32]		+
OASL			[32]		+
OSM			[3,26]	[36]	+
OSMR			[30]		+
PARD3			[30]		+
PDCD1			[30]		+
PDCD1LG2			[30]		+
PDGFB			[11]		+
PER1			[32]		-
PHLPP2			[30]		+
**PI3**			[3,27,32,33]	[3]	+
**PIK3CD**			[30]		+
PIP			[27,32]		-
PKM		[27]			+
PLIN1			[27,48]		+/-
POLR2J2			[3]		-
POU2AF1			[3]		+
**PPARG**			[26]		-
PRDM1			[30]		+
PRKRA			[29]		-
**PSEN1**	[67]				-
PTEN			[30]		+
RASAL3			[3]		+
**RBP4**		[28]			+
RETN		[28]			+
**RORC**			[30]		+
**RPL38**			[3]		-
RPS6			[30]		+
S100A7			[3,30,33,39,42,44,46]	[32]	+
**S100A7A**			[3,27,32]	[3,32]	+
**S100A8**		[57]	[3,33,34,44]	[3,32]	+
**S100A9**		[57]	[3,27,32,33,42,44,46]	[3,32]	+

**S100A12**		[3,30,32,42]	[3,41]	+
SAPCD1		[27]		-
SCGB1D2		[27,32]		-
SCGB2A2		[27,32,33]		-
**SELE**	[54]			+
SEMA3E		[27]		-
SERPINA12		[33]		-
**SERPINB3**		[3,27,30]	[3]	+
SERPINB4		[3,27,30]	[3]	+
**SIRT1**		[26]		-
**SIRT2**			[36]	+
SLAMF7		[3,27]		+
SLC16A3		[3]		+
SLC35E4		[3]		-
SLC66A1		[3]		-
SMPD2		[48]		+
SND1		[29]		-
SOCS1		[26]		-
SP8		[68]		-
SPPL2A		[30]		+
SPRR2B		[32]	[32]	+
SPRR2C		[32]	[32]	+
SPRR2F		[45]		+
SPRR3		[3]	[3]	+
**STAT1**		[3,26,30,44]	[36]	+
STAT2		[30]		+
**STAT3**		[30]		+
**STAT4**		[30]		+
STAT5A		[30]		+
**STAT5B**		[30]		+
STAT6		[30]		+
**SYK**		[30]		+
**TBXA2R**		[3]		-
TCN1		[3,27,45]	[3]	+
TDO2		[27]		+
TENT5C		[33]		+
TGFA		[11]		+
**TGM2**		[26]		+
THRSP		[27]		-
THY1		[3]		+
TLR2		[3,68]		+
**TLR3**		[26]		+
**TLR4**		[26]	[53]	+ / -
TLR5		[3]		+
**TLR9**		[26]		+
TMPRSS11D		[3]	[3]	+
**TNF**		[3,26,30,32,33,38,40]	[56]	+
**TNFRSF1A**		[3]		+
**TNFRSF13B**		[30]		+
TNFRSF14		[30]		+
TNFRSF18		[30]		+
TNFRSF4	[45]	[45]		+
TNFRSF9			[36]	+

TNFSF11		[30]	[36]	+
TNFSF13 (APRIL)		[30]	[26]	+
TNFSF13B (BAFF)		[30]	[26]	+
TNFSF14		[30]	[36]	+
TNFSF18		[30]		+
TNIP1		[26,30]		+ / -
TRIM24		[26]		-
**TRPV1**		[30]		+
**TRPV2**		[30]		+
TSLP		[30]		+
TSPAN8		[27]		-
TTC28		[3]		-
**TYK2**		[30]		+
UCHL1			[69]	-
VAV1		[30]		+
**VEGFA**	[54]			+
WDR97		[3]		-
WIF1		[27,32]		-
**WNT1**		[30]		+
ZFP36		[26]		-
ZNF713		[3]		-
ZSCAN2		[3]		-
ZNF713		[3]		-
ZSCAN2		[3]		-

Table S2. HS-associated DEGs.

Compound	Function	Gene regulation	Development phase
1,2,3,4,5,6-Hexabromocyclohexane	JAK inhibitor	**JAK2**	preclinical
1-EBIO	potassium channel activator	KCNN1, **KCNN2**, KCNN3, KCNN4	preclinical
1-Octanol	Aliphatisches alcohol	GJA1, GJA10, GJA3, GJA4, GJA5, GJA8, GJA9, GJB1, **GJB2**, GJB3, GJB4, GJB5, GJB6, GJB7, GJC1, GJC2, GJC3, GJD2, GJD3, GJD4, GJE1	2
2-Aminobenzenesulfonamide	carbonic anhydrase inhibitor	CA12, CA14, CA2, **CA6**, CA9	preclinical
2-APB	1,4,5-trisphosphate inhibitor	TRPC1, TRPC3, TRPC4, TRPC5, TRPC6, TRPC7, TRPM2, TRPM3, TRPM6, **TRPV1**, TRPV6	preclinical
2,4-Dinitrophenol	ATP synthase inhibitor	**APP**	preclinical
2-Hydroxyflutamide	AR antagonist	**AR**	2
3-Methyladenine	PI3K inhibitor	**PI3**	preclinical
3,3'-Diindolylmethane	CHK inhibitor, cytochrome P450 activator, indoleamine 2,3-dioxygenase inhibitor	**AR**, HIF1A, **IFNG**, **PI3**	3
3,4-Methylenedioxy-β-nitrostyrene	SRC inhibitor, SYK inhibitor	SRC, **SYK**	preclinical
4,5,6,7-Tetrabromobenzotriazole	casein kinase inhibitor	**AKT1**, CHEK1, CSNK2A1, GSK3B, LCK, MAP2K1, MAPK1, MAPK11, MAPK12, MAPK14, MAPK8, PRKCA, ROCK1, RPS6KB1, SGK1	preclinical
6-Iodo-nordihydrocapsaicin	TRPV antagonist	**TRPV1**	preclinical
α-Linolenic acid	ω3 fatty acid stimulant	ELOVL4, FADS1, FADS2, FFAR1, FFAR4, **PTGS2**, SLC8A1, **TRPV1**	3
A-1120	retinoid receptor ligand	**RBP4**	preclinical
A205804	ICAM1 expression inhibitor	ICAM1, **SELE**	preclinical
A-674563	AKT inhibitor	**AKT1**, PKIA, PRKACA	preclinical
A-784168	transient receptor potential channel antagonist	**TRPV1**	preclinical
Abametapir	metalloproteinase inhibitor	**MMP9**	3
Acalabrutinib	Bruton's tyrosine kinase (BTK) inhibitor	**BTK**	launched
Acalisib	PI3K inhibitor	PIK3CB, **PIK3CD**	1
Aceneuramic acid	sialic acid	CES1, **SELE**, SELP	3
Acetohydroxamic acid	urease inhibitor	**MMP12**	launched
Acetyl-farnesyl-cysteine	methyltransferase inhibitor	**PPARG**	launched
Acitretin	retinoid receptor agonist	**KRT16**, **PI3**, RARA, RARB, RARG, RBP1, RXRA, RXRB, RXRG, **STAT3**	launched
Adalimumab	TNF inhibitor	**TNFA**	launched
Adaptavir	CC chemokine receptor antagonist	**CCR5**	2
Adiporon	adiponectin receptor agonist	**ADIPO**R1, **ADIPO**R2	preclinical
AG-490	EGFR inhibitor, JAK inhibitor	**EGF**R, **JAK2**, **JAK3**	preclinical
AK-7	SIRT inhibitor	**SIRT2**	preclinical
AKBA	Lipoxygenase inhibitor	**ALOX5**	3
Alantolactone	apoptosis stimulant, STAT inhibitor	**STAT3**	preclinical
Allicin	cytokine production inhibitor	TRPA1, **TRPV1**	2
Alpelisib	PI3K inhibitor	PIK3CA, PIK3CB, **PIK3CD**, PIK3CG	launched
AMD11070	CC chemokine receptor antagonist	**CCR5**, CXCR4	3

AM-24	lipoxygenase inhibitor	**ALOX5**	2
AM679	cannabinoid receptor agonist, lipoxygenase inhibitor	**ALOX5AP**, CNR1, CNR2	preclinical
AMG-319	PI3K inhibitor	**PIK3CD**	2
Amiloride	sodium channel blocker	AOC1, ASIC1, ASIC2, ASIC3, PKD2, PKD2L1, PLAU, SCNN1A, SCNN1B, SCNN1D, SCNN1G, SLC9A1, TRPC7, **TRPV2**	launched
Aminosalicylate	cyclooxygenase inhibitor	**ALOX5**, CHUK, PLA2G2E, PTGS1, PTGS2	launched
Amlexanox	histamine receptor modulator	**FGF1**, IL3, PDE4A, PDE4B, PDE4C, PDE4D, **S100A12**, S100A13	launched
Amrinone	phosphodiesterase inhibitor	PDE3A, PDE3B, PDE4B, **TNFA**	launched
Anandamide	cannabinoid receptor agonist	CACNA1G, CACNA1H, CACNA1I, CNR1, CNR2, GLRA1, GPR18, GPR55, KCNA2, KCNK3, KCNK9, TRPM8, **TRPV1**	2
Andrographolide	tumor necrosis factor production inhibitor	**IL1B**, **IL6**, **NFKB1**, NFKB2, **TNFA**	2
APcK-110	KIT inhibitor	KIT, **STAT3**	preclinical
API1903	mTOR inhibitor	**MTOR**	1 / 2
API-1	AKT inhibitor	**AKT1**, AKT2, AKT3	preclinical
Apratastat	matrix metalloprotease inhibitor, tumor necrosis factor production inhibitor	ADAM17, **MMP1**, **MMP13**, **MMP9**	2
Apremilast	phosphodiesterase inhibitor	**IFNG**, PDE4A, PDE4B, PDE4C, PDE4D	launched
APY0201	phosphoinositide dependent kinase inhibitor	**IL12A**, **IL12B**, PIKFYVE	preclinical
Arglabin	farnesyltransferase inhibitor	FNTA, **NFKB1**	launched
ARV-825	bromodomain inhibitor	**BRD4**	preclinical
Aspartame	non-saccharide sweetener	TAS1R2, **TRPV1**	launched
Aspirin	cyclooxygenase inhibitor	AKR1C1, ASIC3, EDNRA, HSPA5, IKBKB, **NFKB1**, NFKB2, NFKBIA, PRKAA1, PRKAA2, **PRKAB1**, PRKAB2, PRKAG1, PRKAG2, PRKAG3, **PTGS1**, **PTGS2**, RPS6KA3, TP53	launched
AT13148	Protein kinase inhibitor	**AKT1**, AKT2, AKT3, ROCK1, ROCK2, SGK3	1
AT-9283	Aurora kinase inhibitor, JAK inhibitor	AURKA, AURKB, **JAK2**, **JAK3**, RPS6KA6, STK17A	2
Atiprimod	JAK inhibitor, STAT inhibitor	**JAK2**, **STAT3**	2
Atorvastatin	HMGCR inhibitor	**AHR**	launched
Atractylenolide-i	JAK inhibitor	**JAK1**, **JAK2**, **JAK3**	preclinical
AVL-292	Bruton's tyrosine kinase (BTK) inhibitor	**BTK**, YES1	2
Axitinib	PDGFR tyrosine kinase receptor inhibitor, VEGFR inhibitor	**CSF1**, FLT1, FLT4, KDR, PLK4	launched
AZ20	ATR kinase inhibitor	ATR, **MTOR**	preclinical
AZ876	PPARγ agonist	**PPARG**	preclinical
AZ960	JAK inhibitor	**JAK2**	preclinical
AZD1480	JAK inhibitor	**JAK1**, **JAK2**, **JAK3**	1
AZD2014	mTOR inhibitor	**MTOR**	2
AZD5363	AKT inhibitor	**AKT1**, AKT2, AKT3	3
AZD6482	PI3K inhibitor	PIK3CA, PIK3CB, **PIK3CD**, PIK3CG	1
AZD8055	mTOR inhibitor	**MTOR**	1
AZD8186	PI3K inhibitor	PIK3CB, **PIK3CD**	1

AZD8835	PI3K inhibitor	PIK3CA, **PIK3CD**	1
Baicalein	lipoxygenase inhibitor	**ALOX5**, GLO1, PREP, SELL, SELP, **TNFA**, XDH	preclinical
Balsalazide	cyclooxygenase inhibitor	**ALOX5**, **PPARG**, **PTGS1**, **PTGS2**	launched
Bardoxolone	nuclear factor erythroid derived/like (NRF2) activator	NOS2, **PPARG**	1
Bardoxolone-methyl	nuclear factor erythroid derived/like (NRF2) activator	**PPARG**, **STAT3**	3
Baricitinib	JAK inhibitor	**JAK1**, **JAK2**	launched
Batimastat	matrix metalloprotease inhibitor	ADAM28, ADAMTS5, **MMP12**, MMP16, MMP2, **MMP8**	3
BAY-61-3606	SYK inhibitor	**SYK**	preclinical
BCTC	TRPV antagonist	**TRPV1**	preclinical
Bergenin	interleukin inhibitor	**IL1B**, **TNFA**	preclinical
β-Amyloid-synthesis-inhibitor	β-amyloid synthesis inhibitor	**APP**	preclinical
β-Elemene	apoptosis stimulant	MMP2, **MMP9**	launched
β-hydroxy-beta-Methylbutyrate	protein synthesis stimulant	**MTOR**	launched
Bezafibrate	PPAR agonist	PPARA, PPARD, **PPARG**	launched
BGT226	PI3K inhibitor	**MTOR**, PIK3CA, PIK3CB, PIK3CG	1 / 2
Bicalutamide	AR antagonist	**AR**	launched
BI-2536	PLK inhibitor	**BRD4**, PLK1, PLK2, PLK3	2
Bindarit	NFκB pathway inhibitor	**CCL2**, CCL7, CCL8	2
Bisindolylmaleimide-IX	PKC inhibitor	**AKT1**, GSK3B, LCK, MAPK1, MAPK11, MAPK12, MAPK14, MAPK8, PRKCA, ROCK1, RPS6KB1, SIRT1	preclinical
Bisphenol-A	synthetic estrogen	**AR**, ESR1, ESR2, ESRRG, **PPARG**	1
Bleomycetin	EGFR expression inhibitor	**EGF**R	launched
BMS-536924	IGF-1 inhibitor	**AKT1**, CCNE1, CDK2, **CYP3A4**, ERBB2, IGF1R, KDR, LCK, MAPK1, MET, PDGFRA, PDGFRB	preclinical
BMS-754807	IGF-1 inhibitor	**AKT1**, IGF1R	2
BMS-777607	AXL kinase inhibitor, c-Met inhibitor, FLT3 inhibitor, hepatocyte growth factor receptor inhibitor, macrophage migration inhibiting factor inhibitor, tyrosine kinase inhibitor	AXL, MERTK, MET, **MST1**R, TYRO3	1 / 2
BMS-779788	Liver X receptor agonist	**LXR**	1
BMS-911543	JAK inhibitor	**JAK1**, **JAK2**, **JAK3**	1 / 2
BMS-935177	Bruton's tyrosine kinase (BTK) inhibitor	**BTK**	precinical
BMS-986142	Bruton's tyrosine kinase (BTK) inhibitor	**BTK**	2
BMS-986158	bromodomain inhibitor	**BRD4**	1 / 2
BMS-986195	Bruton's tyrosine kinase (BTK) inhibitor	**BTK**	1
BMS-CCR2-22	CC chemokine receptor antagonist	**CCR2**	preclinical
Brodalumab	IL-17 receptor inhibitor	**IL17R**, **KRT6A**, **S100A7A**, **S100A8**, **S100A9**	launched
BTK-IN-1	Bruton's tyrosine kinase (BTK) inhibitor	**BTK**	preclinical
BW-A4C	lipoxygenase inhibitor	**ALOX5**	1
BW-B70C	lipoxygenase inhibitor	**ALOX5**	preclinical

C-021	CC chemokine receptor antagonist	**CCR4**	preclinical
C34	toll-like receptor inhibitor	**TLR4**	preclinical
Caffeic acid	lipoxygenase inhibitor, tumor necrosis factor production inhibitor	**ALOX5**, MIF	preclinical
Caffeine	adenosine receptor antagonist, phosphodiesterase inhibitor	ADORA1, ADORA2A, ADORA2B, ADORA3, ATM, ITPR1, ITPR2, ITPR3, PDE10A, PDE11A, PDE1A, PDE1B, PDE1C, PDE2A, PDE3A, PDE3B, PDE4A, PDE4B, PDE4C, PDE4D, PDE5A, PDE6A, PDE6B, PDE6C, PDE7A, PDE7B, PDE8A, PDE8B, PDE9A, PIK3CA, PIK3CB, **PIK3CD**, PRKDC, RYR1, RYR2, RYR3	launched
CaMKII-IN-1	calcium/calmodulin dependent protein kinase inhibitor	**AKT1**, CAMK2A, CAMK4, MYLK	preclinical
Canertinib	EGFR inhibitor	**AKT1**, **EGF**R, ERBB2, ERBB4	3
Captopril	angiotensin converting enzyme inhibitor	ACE, **LTA4H**, MMP2, **MMP9**	launched
Carbenoxolone	11-beta hydroxysteroid dehydrogenase inhibitor	GJA1, GJA10, GJA3, GJA4, GJA5, GJA8, GJA9, GJB1, **GJB2**, GJB3, GJB4, GJB5, GJB6, GJB7, GJC1, GJC2, GJC3, GJD2, GJD3, GJD4, GJE1, HSD11B1, PANX1, PANX2, PANX3	launched
Carboxyamidotriazole	calcium channel blocker	**CXCL8**	3
Carzenide	Carbonic anhydrase IX inhibitor	CA1, CA12, CA14, CA2, **CA6**, CA9	preclinical
CB-03-01	AR antagonist	**AR**	3
CBS-1114	lipoxygenase inhibitor	**ALOX5**	1
CC-115	DNA protein kinase inhibitor, mTOR inhibitor	**MTOR**	2
CC-223	mTOR inhibitor	**MTOR**	2
CH223191	Aryl hydrocarbon receptor antagonist	**AHR**	preclinical
CHF5074	γ-secretase modulator	**PSEN1**	2
Chrysophanic acid	EGFR inhibitor	**EGF**R, **MTOR**	preclinical
CEP-33779	JAK inhibitor	**JAK2**	preclinical
Certolizumab pegol	TNF inhibitor	**TNFA**	launched
CH5132799	PI3K inhibitor	**MTOR**, PIK3CA, PIK3CB, **PIK3CD**, PIK3CG	1
Cholic acid	bile acid synthesis disorders, peroxisomal disorders	**ADH1C**	launched
Ciglitazone	PPARγ agonist	**GPD1**, **PPARG**, **TBXA2R**	2
CINPA-1	CAR antagonist	**NR1H4**, NR1I3, **PPARG**	preclinical
Cipemastat	metalloproteinase inhibitor	ADAM17, **MMP1**, MMP13, MMP2, **MMP3**, **MMP8**, **MMP9**	3
Citric acid	coagulation factor inhibitor	AKR1B1, ANG, APRT, BHMT, C8G, CA4, CPB1, CS, CTDSP1, GNMT, HGS, HS3ST3A1, IL4I1, ITPA, LSM6, MDH2, MIF, PDE5A, PKD2L1, PLEKHA1, RNASE1, RNASE3, SRC, **TNFSF13B,** UCK2	preclinical
CKD-712	NFκB pathway inhibitor	**JAK2**, **STAT1**	1
Cloprostenol-(+/-)	prostaglandin receptor agonist	PTGDR, PTGER1, PTGER3, PTGFR, **TBXA2R**	launched
CNX-774	Bruton's tyrosine kinase (BTK) inhibitor	**BTK**	preclinical

Coenzyme-I		AASS, ACADS, ADH1A, ADH1B, **ADH1C**, ADH4, ADH5, ADH7, AKR1B1, AKR1C1, AKR1C2, AKR1C3, AKR1C4, ALDH1A1, ALDH1A2, ALDH1A3, ALDH1B1, ALDH2, ALDH3A1, ALDH3A2, ALDH3B1, ALDH3B2, ALDH4A1, ALDH5A1, ALDH6A1, ALDH7A1, ALDH9A1, AMT, BDH1, BLVRA, BLVRB, CDO1, CYB5R3, CYP17A1, CYP4A11, DHCR7, DHFR, DLAT, DLD, EHHADH, **GAPDH**, GAPDHS, GLUD1, GLUD2, **GPD1**, GSR, H6PD, HADH, HADHA, HIBADH, HMGCR, HMOX1, HMOX2, HPGD, HSD11B1, HSD11B2, HSD17B1, HSD17B10, HSD17B2, HSD17B3, HSD17B4, HSD17B7, HSD17B8, HSD3B1, HSD3B2, IDH3A, IDH3B, IDH3G, IMPDH1, IMPDH2, LDHA, LDHAL6A, LDHAL6B, LDHB, LDHC, MDH1, MDH2, ME1, ME2, ME3, MSMO1, MT-ND1, MT-ND2, MT-ND3, MT-ND4, MT-ND4L, MT-ND5, MT-ND6, MTHFD1, MTHFD2, NDUFA1, NDUFA10, NDUFA11, NDUFA12, NDUFA13, NDUFA2, NDUFA3, NDUFA4, NDUFA4L2, NDUFA5, NDUFA6, NDUFA7, NDUFA8, NDUFA9, NDUFAB1, NDUFB1, NDUFB10, NDUFB2, NDUFB3, NDUFB4, NDUFB5, NDUFB6, NDUFB7, NDUFB8, NDUFB9, NDUFC1, NDUFC2, NDUFS1, NDUFS2, NDUFS3, NDUFS4, NDUFS5, NDUFS6, NDUFS7, NDUFS8, NDUFV1, NDUFV2, NDUFV3, NNT, NQO2, NSDHL, OGDH, PDHA1, PDHA2, PDHB, PHGDH, PYCR1, PYCR2, QDPR, RDH5, SORD, TSTA3, TYR, UGDH	2
Compound-401	DNA dependent protein kinase inhibitor	**MTOR**, PRKDC	preclinical
Copanlisib	PI3K inhibitor	PIK3CA, PIK3CB, **PIK3CD**, PIK3CG	launched
CP-471474	matrix metalloprotease inhibitor	**MMP1**, MMP13, MMP2, **MMP3**, **MMP9**	preclinical
CPI-1189	tumor necrosis factor release inhibitor	**TNFA**	2
Cortodoxone	AR antagonist	**AR**	3
Coumarin	vitamin K antagonist	CA1, CA12, CA14, CA2, CA4, **CA6**, CA9, CYP2A6	launched
CPI-0610	bromodomain inhibitor	**BRD4**	2
CPI-203	bromodomain inhibitor	**BRD4**	preclinical
Cryptotanshinone	acetylcholinesterase inhibitor, STAT inhibitor	**STAT3**	preclinical
CTS-1027	metalloproteinase inhibitor	**MMP1**, MMP13, MMP2, **MMP3**, **MMP9**	2
CU-CPT-4a	toll-like receptor inhibitor	**TLR3**	preclinical
Curcumin	cyclooxygenase inhibitor, histone acetyltransferase inhibitor, lipoxygenase inhibitor, NFκB pathway inhibitor	**APP**, CA1, CA12, CA14, CA2, CA4, **CA6**, CA9, CHRM3, **CYP3A4**, DNMT3B, EP300, MMP13, **MMP9**, NOS2, **PTGS1**, **PTGS2**, XDH	launched

Curcumol	JAK inhibitor	**JAK1**, **JAK2**, **JAK3**	1
Cyproterone acetate	AR antagonist	ADORA1, **AR**	launched
Cyt387	JAK inhibitor	**JAK1**, **JAK2**, **JAK3**	3
Danazol	estrogen receptor antagonist, progesterone receptor agonist	**AR**, **CCL2**, ESR1, GNRHR, GNRHR2, PGR	launched
Darglitazone	PPARγ agonist	**PPARG**	2
Darolutamide	AR antagonist	**AR**	launched
Dasatinib	Bcr-Abl kinase inhibitor, ephrin inhibitor, KIT inhibitor, PDGFR tyrosine kinase receptor inhibitor, SRC inhibitor, tyrosine kinase inhibitor	ABL1, ABL2, BLK, EPHA2, **FGR**, FRK, FYN, HCK, KIT, LCK, LYN, PDGFRB, SRC, SRMS, **STAT5B**, YES1	launched
DCEBIO	potassium channel activator	**KCNN2**, KCNN3, KCNN4	preclinical
D-serine	glutamate receptor agonist	GLRA1, GRIN1, GRIN2A, GRIN2B, GRIN2C, GRIN2D, **SERPINB3**	launched
Decernotinib	JAK inhibitor	**JAK3**	2 / 3
Deforolimus	mTOR inhibitor	**MTOR**	3
Delgocitinib	JAK inhibitor	**JAK1**, **JAK2**, **JAK3**	2
Dexfosfoserine	membrane integrity inhibitor	CFTR, **GRM4**, GRM6, GRM7, GRM8, KCNC4, PDPK1, PIM1, PRKACA, PRKCQ, PYGL, PYGM, REG1A, RHO, **SERPINB3**, SMAD2, TAOK2	preclinical
Diacerein	interleukin inhibitor	**IL1B**	launched
Diclofenac	cyclooxygenase inhibitor	AKR1C3, **ALOX5**, ASIC1, ASIC3, KCNQ2, KCNQ3, PLA2G2A, **PPARG**, **PTGS1**, **PTGS2**, SCN4A	launched
Diethylcarbamazine	lipoxygenase inhibitor	**ALOX5**, PTGS1	launched
Dinoprost	prostacyclin analog	PTGDR, PTGDR2, PTGER1, PTGER2, PTGER3, PTGER4, PTGFR, **TBXA2R**	launched
Docebenone	lipoxygenase inhibitor	**ALOX5**	2
Doxycycline	bacterial 30S ribosomal subunit inhibitor, metalloproteinase inhibitor	**MMP1**, **MMP8**, **PI3**	launched
Dupilumab	interleukin receptor antagonist	**IL4R**	launched
Duvelisib	PI3K inhibitor	PIK3CA, PIK3CB, **PIK3CD**, PIK3CG	launched
Edaglitazone	PPARγ agonist	**PPARG**	2
Efatutazone	PPARγ agonist	**PPARG**	2
Elafibranor	PPAR agonist	PPARA, PPARD, **PPARG**	3
Ellagic acid	glutathione transferase inhibitor, non-nucleoside reverse transcriptase inhibitor	CA1, CA12, CA14, CA2, CA3, CA4, CA5A, CA5B, **CA6**, CA7, CA9, CSNK2A1, GSK3B, PRKACA, PRKCA, PRKCB, SQLE, **SYK**	2
Emricasan	caspase inhibitor	**CASP1**, CASP3, CASP7	2
Enzalutamide	AR antagonist	**AR**	launched
Enzastaurin	PKC inhibitor	**AKT1**, GSK3B, PRKCA, PRKCB, PRKCD, PRKCG	3
Epinephrine	adrenergic receptor agonist, carbonic anhydrase activator, neurotransmitter	**ADRA1A**, ADRA1B, ADRA1D, ADRA2A, ADRA2B, ADRA2C, ADRB1, ADRB2, ADRB3, PAH, **TNFA**	launched
Ephedrine (racemic)	adrenergic receptor agonist	**ADRA1A**, ADRA2A, ADRB1, ADRB2, ATF1, ATF2, ATF3, ATF4, ATF5, ATF6, ATF7, FOS, IL2, JDP2, JUN, NFATC1, SLC6A2, SLC6A3, **SLC6A4**, **TNFA**	launched
ER-27319	mediator release inhibitor, SYK inhibitor	**SYK**	preclinical
Erlotinib	EGFR inhibitor	**EGFR**, NR1I2	launched

Etanercept	TNF-α receptor antagonist	**TNFAR**	launched
Ethanolamine-oleate	immunostimulant	F12, **FABP4**, FFAR1, FFAR4, PLD2	launched
Ethoxzolamide	carbonic anhydrase inhibitor	CA1, CA12, CA13, CA14, CA2, CA3, CA4, CA5A, CA5B, **CA6**, CA7, CA9	launched
ETP-45658	PI3K inhibitor	PIK3CA, PIK3CB, **PIK3CD**, PIK3CG	preclinical
Eugenol	AR antagonist	**AR**	launched
Everolimus	mTOR inhibitor	**MTOR**	launched
Evobrutinib	Bruton's tyrosine kinase (BTK) inhibitor	**BTK**	2
Fedratinib	FLT3 inhibitor, JAK inhibitor	**BRD4**, **JAK1**, **JAK2**, **JAK3**, **TYK2**	launched
Fenebrutinib	Bruton's tyrosine kinase (BTK) inhibitor	**BTK**	2
Ferulic acid	Antioxidant	CA1, CA12, CA14, CA2, CA4, **CA6**, CA9	2
Filgotinib	JAK inhibitor	**JAK1**, **JAK2**, **JAK3**, **TYK2**	3
Flufenamic acid	chloride channel blocker	AKR1C3, ANO1, **AR**, GJA1, GJA10, GJA3, GJA4, GJA5, GJA8, GJA9, GJB1, **GJB2**, GJB3, GJB4, GJB5, GJB6, GJB7, GJC1, GJC2, GJC3, GJD2, GJD3, GJD4, GJE1, PANX1, PANX2, PANX3, PKD2L1, **PTGS1**, **PTGS2**, TRPC5, TRPM2, TRPM5	preclinical
Flutamide	Androgen receptor antagonist	**AR**, **AHR**	launched
Fostamatinib	SYK inhibitor	**SYK**	launched
Gallic acid	beta-amyloid protein neurotoxicity inhibitor, PPAR agonist	CA1, CA12, CA14, CA2, CA4, **CA6**, CA9, SELP	preclinical
Ganoderic-acid-a	JAK inhibitor	**JAK1**, **JAK2**, **JAK3**	preclinical
GDC-0068	AKT inhibitor	**AKT1**, AKT2, AKT3, PRKG1	3
GDC-0834	Bruton's tyrosine kinase (BTK) inhibitor	**BTK**	1
GDC-0941	PI3K inhibitor	PIK3CA, PIK3CB, **PIK3CD**, PIK3CG	2
GDC-0980	mTOR inhibitor, PI3K inhibitor	**FGR**, MAP3K9, **MTOR**, PIK3CA, PIK3CB, **PIK3CD**, PIK3CG, **SYK**	2
Gefitinib	EGFR inhibitor	**EGFR**	launched
Genistein	tyrosine kinase inhibitor	**CFD**, CFT, **DEFB4B**, ESR1, ESR2, ESRRA, ESRRB, ESRRG, **GJB2**, **MMP1**, NCOA1, NCOA2, **PI3**, **PPARG**, PTK2B, TOP2A, TRPC5	3
GK921	transglutaminase inhibitor	**TGM2**	preclinical
Glipizide	sulfonylurea	ABCC8, KCNJ10, KCNJ11, **PPARG**	launched
Glycitein	tumor necrosis factor release inhibitor	**TNFA**	3
Glucosamine	glycosylated protein precursor	**IL1B**	launched
Golimumab	TNF inhibitor	**TNFA**	launched
GS-9973	SYK inhibitor	**SYK**	2
GSK2110183	AKT inhibitor	**AKT1**, AKT2, AKT3	2
GSK2126458	mTOR inhibitor, PI3K inhibitor	**MTOR**, PIK3CA, **PIK3CB**, PIK3CD, PIK3CG	1
GSK2190915	lipoxygenase inhibitor	**ALOX5AP**	2
GSK690693	AKT inhibitor	**AKT1**, AKT2, AKT3, PAK4P, PAK6, PAK7, PRKCQ, PRKG1, PRKX	1
GW-1929	PPARγ agonist	**PPARG**	preclinical
GW-3965	Liver X receptor agonist	**NR1H2**, **NR1H3**	preclinical
Halofuginone	collagenase inhibitor	COL1A1, **MMP2**	launched
Hemomex-s	PPARγ agonist	**PPARG**	launched

Hexamethylenebisacetamide	AKT inhibitor, differentiation inducer, NFκB pathway inhibitor	**AKT1**	2
Honokiol	AKT inhibitor	**ALOX5**, PTGS1, PTGS2	3
Hydroxychloroquine	antimalarial agent	TLR7, **TLR9**	launched
Hypoestoxide	IKK inhibitor	IKBKB, **IL1B**, **TNFA**	1
I-BET151	bromodomain inhibitor	BRD2, BRD3, **BRD4**	preclinical
I-BET-762	bromodomain inhibitor	BRD2, BRD3, **BRD4**	2
Ibrutinib	Bruton's tyrosine kinase (BTK) inhibitor	BLK, BMX, **BTK**	launched
Ibudilast	leukotriene receptor antagonist, phosphodiesterase inhibitor	**IL1B**, **IL6**, PDE3A, PDE4A, PDE4B, PDE4C, PDE4D, PDE5A	launched
Ibuproxam	cyclooxygenase inhibitor, prostaglandin inhibitor	**ALOX5**	launched
Icosapent	platelet aggregation inhibitor	ACSL3, ACSL4, FADS1, FFAR1, PPARD, **PPARG**, **PTGS1**, **PTGS2**, SLC8A1, **TRPV1**	launched
Icotinib	EGFR inhibitor	**EGFR**	launched
ID-1101	insulin sensitizer	**INS**	1
Idelalisib	PI3K inhibitor	PIK3CA, PIK3CB, **PIK3CD**, PIK3CG	launched
Ilomastat	matrix metalloprotease inhibitor	CAN, ADAM28, **MMP1**, **MMP12**, MMP13, MMP14, MMP2, **MMP3**, **MMP8**, **MMP9**	3
Iloprost	platelet aggregation inhibitor, prostanoid receptor agonist	PTGDR, PTGER1, PTGER2, PTGER3, PTGER4, PTGFR, PTGIR, **TBXA2R**	launched
INCB-3284	CC chemokine receptor antagonist	**CCR2**	2
Indeglitazar	PPAR agonist	NCOA1, PPARA, PPARD, **PPARG**	2
Indisulam	CDK inhibitor	CA1, CA12, CA14, CA2, **CA6**, CA7, CA9	2
Indomethacin	cyclooxygenase inhibitor	GLO1, PLA2G2A, PPARA, **PPARG**, PTGDR2, PTGR2, **PTGS1**, **PTGS2**, SLC46A1	launched
Infliximab	TNF inhibitor	**IL6**, **TNFA**	launched
Itacitinib	JAK inhibitor	**JAK1**	3
JAK3-inhibitor-V	JAK inhibitor	**JAK3**	preclinical
JNJ-17203212	TRPV antagonist	**TRPV1**	preclinical
JNJ-27141491	CC chemokine receptor antagonist	**CCR2**	preclinical
JQ1-(+)	bromodomain inhibitor	**BRD4**, BRDT	preclinical
JTE-607	cytokine production inhibitor	**IL10**, **IL1B**, **IL6**, **TNFA**	2
JW-67	WNT pathway inhibitor	APC, **AXIN1**, GSK3B	preclinical
KN-62	calcium/calmodulin dependent protein kinase inhibitor, purinergic receptor antagonist	**AKT1**, CAMK2A, CHEK1, LCK, MAPK1, MAPK11, MAPK12, MAPK14, MAPK8, P2RX7, PRKCA, ROCK1, RPS6KB1, SGK1	preclinical
KU-0063794	mTOR inhibitor	**MTOR**	preclinical
L-alanine	Non-essential aminoacid	AARS, AARS2, **ABAT**, AGXT, AGXT2, GPRC6A, GPT, GPT2, **KYNU**, NFS1, PHYKPL, SLC1A4, SLC36A1, SLC7A8	launched
Laropiprant	prostanoid receptor antagonist	PTGDR, PTGDR2, PTGER1, PTGER2, PTGER3, PTGFR, PTGIR, **TBXA2R**	launched
Latamoxef	cephalosporine	**DACB**, **MRCA**, **MRCB**, **PBPC**	launched
Lauric acid	bacterial permeability inducer	GPR84, HNF4A, LTF, LY96, PLA2G2A, **TLR4**	3

L-cysteinesulfinic acid	glutamate receptor agonist	GRM1, GRM5, GSR, **HMGCS2**, PAPOLA, PRDX2	preclinical
LDN-27219	transglutaminase inhibitor	**TGM2**	preclinical
Leflunomide	dihydroorotate dehydrogenase inhibitor, PDGFR tyrosine kinase receptor inhibitor	**AHR**, DHODH, PTK2B	launched
Lenalidomide	tumor necrosis factor production inhibitor	**TNFA**	launched
LFM-A13	Bruton's tyrosine kinase (BTK) inhibitor	**BTK**	preclinical
Licofelone	cyclooxygenase inhibitor, lipoxygenase inhibitor	**ALOX5**, PLA2G2E, PTGS2	3
Ligustilide	tumor necrosis factor production inhibitor	**TNFA**	preclinical
Linifanib	PDGFR tyrosine kinase receptor inhibitor, VEGFR inhibitor	**CSF1**, CSF1R, FLT1, FLT3, FLT4, KDR, KIT, PDGFRB, RET, TEK	3
Linoleic acid	oxidative stress inducer	**FABP4**, FFAR, FFAR4, HNF4A, KCNB1, **PPARG**, TRPM8	3
Lupanine	sodium channel blocker	**INS**	1
LXR-623	Liver X receptor agonist	**AR**, **NR1H2**, **NR1H3**, NR1I2, NR3C1	1
LY2784544	JAK inhibitor	**JAK2**, **JAK3**	2
LY294002	DNA dependent protein kinase inhibitor, mTOR inhibitor, phosphodiesterase inhibitor, PI3K inhibitor, PLK inhibitor	**AKT1**, CHEK1, GSK3B, LCK, MAPK1, MAPK11, MAPK12, MAPK14, MAPK8, **MTOR**, PIK3CA, PIK3CB, **PIK3CD**, PIK3CG, PIM1, PLK1, PRKCA, PRKDC, ROCK1, RPS6KB1, SGK1	preclinical
LY3023414	mTOR inhibitor, PI3K inhibitor	**MTOR**	2
LY303511	casein kinase inhibitor, mTOR inhibitor, PI3K inhibitor	BRD2, BRD3, **BRD4**	preclinical
Mafenide	carbonic anhydrase inhibitor	CA12, CA14, CA2, CA4, **CA6,** CA9	launched
Magnolol	PPARγ agonist	GABRA1, **PPARG**	preclinical
Maraviroc	CC chemokine receptor antagonist	**CCR5**	launched
Marimastat	matrix metalloprotease inhibitor	**MMP1**, **MMP10**, MMP11, **MMP12**, MMP13, MMP14, MMP15, MMP16, MMP17, MMP19, MMP2, MMP20, MMP21, MMP23A, MMP24, MMP25, MMP26, MMP27, MMP28, **MMP3**, MMP7, **MMP8**, **MMP9**	3
MC-1	purinergic receptor inhibitor, intracellular Ca^{2+} influx inhibitor	AADAT, ABAT, AGXT, AGXT2, ALAS1, AZIN2, BCAT1, BCAT2, CBS, CCBL1, CCBL2, CSAD, CTH, DDC, FTCD, GAD1, GAD2, GADL1, GCAT, GLDC, GOT1, GOT2, GPT, GPT2, HDC, IGSF10, **KYNU**, MOCOS, NFS1, OAT, ODC1, PDXDC1, PDXP, PHYKPL, PNPO, PROSC, PSAT1, PYGB, PYGL, PYGM, SCLY, SDS, SDSL, SEPSECS, SGPL1, SHMT1, SHMT2, SPTLC1, SPTLC2, SPTLC3, SRR, TAT, THNSL1	3
Masoprocol	lipoxygenase inhibitor	**ALOX5**	launched
MCC950	NOD like receptor inhibitor	**NLRP3**	preclinical
Meclofenamic acid	cyclooxygenase inhibitor, prostanoid receptor antagonist	**ALOX5**, CNR1, KCNQ2, KCNQ3, PTGS1, PTGS2	launched
Meglitinide	potassium channel blocker	**CCR2**	2
Meisoindigo	STAT inhibitor	**STAT3**	3

Mesalazine	cyclooxygenase inhibitor, lipoxygenase inhibitor, prostanoid receptor antagonist	**ALOX5**, CHUK, IKBKB, MPO, **PPARG**, **PTGS1**, **PTGS2**	launched
Metacresol	phenol derivative	**INS**	launched
Metformin	insulin sensitizer	ACACB, **PRKAB1**	launched
Mimosine	DNA replication inhibitor	**CCL2**, SHMT1, SHMT2, TYR	preclinical
Mitiglinide	insulin secretagogue	ABCC8, KCNJ10, **PPARG**	launched
MK-0812	CC chemokine receptor antagonist	**CCR2**	2
MK-2206	AKT inhibitor	**AKT1**, AKT2, AKT3	2
MK-2295	TRPV antagonist	**TRPV1**	2
MK-2461	FGFR inhibitor, VEGFR inhibitor	FGFR1, FGFR2, FGFR3, FLT1, FLT3, FLT4, KDR, MERTK, MET, **MST1**R, NTRK1, NTRK2, PDGFRB	1 / 2
MLN0128	mTOR inhibitor	**MTOR**, PIK3CA, **PIK3CD**, PIK3CG	2
Monepantel	mTOR inhibitor	**MTOR**	preclinical
Montelukast	leukotriene receptor antagonist	**ALOX5**, CYSLTR1	launched
MRK-560	γ-secretase inhibitor	**APP**	preclinical
Naloxone	opioid receptor antagonist	CREB1, ESR1, **OPRD1**, OPRK1, **OPRM**, **TLR4**	launched
Napabucasin	STAT inhibitor	**STAT3**	3
Nateglinide	insulin secretagogue	ABCC8, KCNJ10, KCNJ11, **PPARG**	launched
Niclosamide	DNA replication inhibitor, STAT inhibitor	**STAT3**	launched
Nilutamide	AR antagonist	**AR**	launched
NLG919	indoleamine 2,3-dioxygenase inhibitor IDO1	**IDO1**	1
NDT-9513727	complement antagonist	**C5AR1**	preclinical
NS-018	JAK inhibitor	**JAK1**, **JAK2**, **JAK3**, **TYK2**	1 / 2
NS-309	calcium-activated potassium channel activator	KCNN1, **KCNN2**, KCNN3, KCNN4	preclinical
NS-8593	calcium-activated potassium channel modulator	KCNN1, **KCNN2**, KCNN3	preclinical
NSC-405020	matrix metalloprotease inhibitor	**MMP1**	preclinical
nTZDpa	PPARγ agonist	**PPARG**	preclinical
NVP-BEZ235	mTOR inhibitor, PI3K inhibitor	ATR, **MTOR**, PIK3CA, **PIK3CD**, PIK3CG	3
NVP-BSK805	JAK inhibitor	**JAK2**	preclinical
Ochromycinone	STAT inhibitor	**STAT3**	2
Olmutinib	Bruton's tyrosine kinase (BTK) inhibitor, EGFR inhibitor	**BTK**, **EGF**R	launched
Olopatadine	histamine receptor antagonist	**HRH1**, S100A1, **S100A12**, S100A13, S100A2, S100B	launched
Olsalazine	cyclooxygenase inhibitor	**IFNG**, TPMT	launched
ONO-4059	Bruton's tyrosine kinase (BTK) inhibitor	**BTK**	1
ONO-4817	matrix metalloprotease inhibitor	**MMP8**	1
OSI-027	mTOR inhibitor	**MTOR**	1
Osimertinib	EGFR inhibitor	**EGF**R	launched
OTX015	bromodomain inhibitor	BRD2, BRD3, **BRD4**	1 / 2
OXF-BD-02	bromodomain inhibitor	**BRD4**	preclinical
Pacritinib	FLT3 inhibitor, JAK inhibitor	FLT3, **JAK1**, **JAK2**, **JAK3**	3
Palomid-529	AKT inhibitor, mTOR inhibitor	**MTOR**	1
Paquinimod	S100A9 inhibitor	**S100A9**	2
Para-toluenesulfonamide	G1 cell cycle arrest inducer	CA12, CA2, **CA6**, CA9	3

Paclitaxel	tubulin polymerization inhibitor	BCL2, MAP2, MAP4, MAPT, NR1I2, **TLR4**, TUBA1A, TUBA1B, TUBA1C, TUBA3C, TUBA3D, TUBA3E, TUBA4A, TUBB, TUBB1, TUBB2A, TUBB2B, TUBB3, TUBB4A, TUBB4B, TUBB6, TUBB8	launched
Paracetamol	cyclooxygenase inhibitor	FAAH, **PTGS1**, **PTGS2**, **TRPV1**	launched
Pazopanib	KIT inhibitor, PDGFR tyrosine kinase receptor inhibitor, VEGFR inhibitor	CSF1R, **FGF1,** FGFR1, FGFR3, FLT1, FLT4, **ITK**, KDR, KIT, PDGFRA, PDGFRB, SH2B3	launched
PCI-29732	Bruton's tyrosine kinase (BTK) inhibitor	**BTK**	preclinical
PD1-PDL-inhibitor-1	programmed death ligand inhibitor	**CD274**	preclinical
PD-166793	collagenase inhibitor, metalloproteinase inhibitor	MMP13, MMP2, **MMP3**	preclinical
PD-98059	MEK inhibitor	**AKT1**, CHEK1, GSK3B, LCK, MAP2K1, MAPK1, MAPK11, MAPK12, MAPK14, MAPK8, PRKCA, RAF1, ROCK1, RPS6KB1, SGK1	preclinical
Peficitinib	JAK inhibitor	**JAK1**, **JAK2**, **JAK3**	launched
Pentoxifylline	phosphodiesterase inhibitor	ADORA, ADORA2A, ADORA2B, ADRA2B, NT5E, PDE10A, PDE1A, PDE1B, PDE1C, PDE2A, PDE3A, PDE3B, PDE4A, PDE4B, PDE4C, PDE4D, PDE5A, PDE6A, PDE6B, PDE6C, PDE6D, PDE6G, PDE6H, PDE7A, PDE7B, PDE8A, PDE8B, PDE9A, **TNFA**	launched
Perifosine	AKT inhibitor	**AKT1**	3
PF-04691502	mTOR inhibitor, PI3K inhibitor	**MTOR**, PIK3CA	2
PF-05212384	mTOR inhibitor, PI3K inhibitor	**MTOR**, PIK3CA	2
PF-06651600	JAK inhibitor	**JAK1, JAK2, JAK3**	2 / 3
PFI-1	bromodomain inhibitor	**BRD4**	preclinical
PI-103	mTOR inhibitor, PI3K inhibitor	**MTOR**, PIK3CA, PIK3CB, **PIK3CD**, PIK3CG, PRKDC	preclinical
PIK-293	PI3K inhibitor	PIK3CA, PIK3CB, **PIK3CD**, PIK3CG	preclinical
PIK-294	PI3K inhibitor	PIK3CB, **PIK3CD**, PIK3CG	preclinical
PIK-75	DNA protein kinase inhibitor, PI3K inhibitor	PIK3CA, PIK3CB, **PIK3CD**, PIK3CG, PRKDC	preclinical
Pioglitazone	insulin sensitizer, PPARγ agonist	**PPARG**, **ADIPOQ**, TRPM3	launched
Pirfenidone	TGF beta receptor inhibitor	FURIN, **TNFA**	launched
PKI-179	mTOR inhibitor, PI3K inhibitor	**MTOR**	1
Plerixafor	CC chemokine receptor antagonist	ACKR3, **CCR4**, CXCR4, **MMP1**, **PI3**	launched
PP-121	protein tyrosine kinase inhibitor	ABL1, **EGFR**, HCK, KDR, **MTOR**, PDGFRA, PIK3CA, PIK3CB, **PIK3CD**, PIK3CG, PRKDC, SRC	preclinical
PP242	mTOR inhibitor	**MTOR**, PASK	preclinical
Pranlukast	leukotriene receptor antagonist	CYSLTR1, CYSLTR2, **IL5**, MUC2, **NFKB1**, RNASE3, **TNFA**	launched
PRN1008	Bruton's tyrosine kinase (BTK) inhibitor	**BTK**	preclinical
PRT062070	JAK inhibitor, SYK inhibitor	FGR, MAP3K9, **SYK**	2 / 3
PRT062607	SYK inhibitor	**SYK**	2
Pyrvinium pamoate	AR antagonist	**AR**	launched

Quiflapon	leukotriene synthesis inhibitor	**ALOX5**, **ALOX5AP**	2
R112	SYK inhibitor	**SYK**	1
R406	SYK inhibitor	RET, **SYK**	1
Repaglinide	insulin secretagogue	ABCC8, KCNJ11, **PPARG**	launched
Resatorvid	toll-like receptor inhibitor	**TLR4**	3
REV-5901	leukotriene receptor antagonist, lipoxygenase inhibitor	**ALOX5**	2
RGB-286638	CDK inhibitor	CDK1, CDK2, CDK3, CDK4, CDK5, CDK6, CDK7, CDK9, FLT3, GSK3B, **JAK2**, MAP3K7, MAPK9	1
RGX-104	Liver X receptor agonist	**LXR**	1
Ro-90-7501	β-amyloid protein neurotoxicity inhibitor	**APP**	preclinical
Rovazolac	liver X receptor agonist	**LXR**	preclinical
RS-102895	CCR antagonist	**CCR2**	preclinical
RS-504393	CC chemokine receptor antagonist	**CCL2**, **CCR2**	preclinical
RU-58841	AR inhibitor	**AR**	2
Ruxolitinib	JAK inhibitor	**JAK1**, **JAK2**, **JAK3**, **TYK2**	launched
Ruxolitinib-(S)	JAK inhibitor	**JAK1**, **JAK2**	preclinical
S26948	PPARγ agonist	**PPARG**	preclinical
Saroglitazar	PPAR agonist	PPARA, **PPARG**	launched
Salvianolic acid-B	EGFR inhibitor, metalloproteinase inhibitor	**MMP9**	2
SAR-245409	PI3K inhibitor	**MTOR**, PIK3CG	1 / 2
SB-202190	p38 MAPK inhibitor	AKT1, **ALOX5**, CHEK1, GSK3B, LCK, MAPK1, MAPK11, MAPK12, MAPK14, MAPK8, PRKCA, ROCK1, RPS6KB1, SGK1	preclinical
SB-203580	p38 MAPK inhibitor	**AKT1**, **ALOX5**, CHEK1, CYP2D6, **CYP3A4**, GAK, GSK3B, LCK, MAPK1, MAPK10, MAPK11, MAPK12, MAPK14, MAPK8, MAPK9, PRKCA, RAF1, RIPK2, ROCK1, RPS6KB1, SGK1, SRC, **TNFA**	preclinical
SB-2343	mTOR inhibitor, PI3K inhibitor	**MTOR**, PIK3CA, PIK3CB, **PIK3CD**, PIK3CG	1
SB-366791	TRPV antagonist	**TRPV1**	preclinical
SB-452533	TRPV antagonist	**TRPV1**	preclinical
SB-705498	TRPV antagonist	**TRPV1**	preclinical
SB-747651A	kinase inhibitor	**AKT1**, AKT3, ROCK1, RPS6KA5, RPS6KB1	preclinical
SC-12267	dihydroorotate dehydrogenase inhibitor	**IL17A**	2
SD-2590	matrix metalloprotease inhibitor	**MMP1**, MMP13, MMP2	preclinical
SEN-1269	β-amyloid inhibitor	**APP**	preclinical
Silibinin	cytochrome P450 inhibitor	**ALOX5**	launched
Sirolimus	mTOR inhibitor	**CFD1**, FKBP1A, **GPD1**, **MMP1**, **MTOR**, **PI3**, **RPL38**	launched
SirReal-2	SIRT inhibitor	**SIRT2**	preclinical
SKF-86002	p38 MAPK inhibitor	**ALOX5**, MAPK14	preclinical
SKF-96365	calcium channel blocker	**CYP3A4**, PKD2, TRPC1, TRPC3, TRPC4, TRPC5, **TRPV2**	preclinical
Solcitinib	JAK inhibitor	**JAK1**	1
SP-100030	NFkB pathway inhibitor	JUN, **NFKB1**	preclinical

Spermine	Cellular metabolism	CA14, CA4, **CA6**, CA9, CASR, GRIN1, GRIN2A, KCNJ4, ODC1, SMOX, SMS, TRPM4, TRPM5	preclinical
SR-1664	PPAR ligand	**PPARG**	preclinical
SR-2211	retinoid receptor inverse agonist	**RORC**	preclinical
SRT1720	SIRT activator	**SIRT1**	preclinical
SRT2104	SIRT activator	**SIRT1**	2
ST-2825	myeloid differentiation primary response inhibitor	**MYD88**	preclinical
STA-5326	interleukin synthesis inhibitor	**IL12A**	2
STAT3 inhibitor-VI	STAT inhibitor	**STAT3**	preclinical
Stemregenin-1	Aryl hydrocarbon receptor antagonist	**AHR**	preclinical
Sulfasalazine	cyclooxygenase inhibitor	ACAT1, **ALOX5**, CHUK, IKBKB, PLA2G1B, **PPARG**, **PTGS1**, **PTGS2**, SLC46A1, SLC7A11, TBXAS1	launched
T-0901317	Liver X receptor agonist	NCOA1, NCOA2, **NR1H2**, **NR1H3**, NR1I2, RXRB	preclinical
TAK-220	CC chemokine receptor antagonist	**CCR5**	1
Talmapimod	p38 MAPK inhibitor	**IL1B**, MAPK11, MAPK14, MT-CO2, **TNFA**	2
Tanshinone-I	AP inhibitor	**IFNG**	2
Tanshinone-IIA	anti-inflammatory agent,interleukin inhibitor	**IL1B**, NR1I2, **TNFA**	2 / 3
Tapinarof	aryl hydrocarbon receptor agonist	**AHR**	3
Taxifolin	opioid receptor antagonist	**ADIPO**R2	2
TCS-21311	JAK inhibitor	**JAK3**	preclinical
Teijin compound-1	CC chemokine receptor antagonist	**CCR2**	preclinical
Telmisartan	angiotensin receptor antagonist	AGTR1, **PPARG**	launched
Temsirolimus	mTOR inhibitor	**MTOR**	launched
Tepoxalin	cyclooxygenase inhibito, lipoxygenase inhibitor	**ALOX5**	launched
Terreic-acid-(-)	Bruton's tyrosine kinase (BTK) inhibitor	**BTK**	preclinical
Tesaglitazar	insulin sensitizer, PPAR agonist	PPARA, **PPARG**	3
Thalidomide	tumor necrosis factor production inhibitor	**HGF**, **TNFA**	launched
Thiram	JAK inhibitor	**JAK2**	launched
TG-02	CDK inhibitor, FLT3 inhibitor, JAK inhibitor	CDK1, CDK2, CDK7, CDK9, FLT3, **JAK2**	1 / 2
TG100-115	PI3K inhibitor	PIK3CA, PIK3CB, **PIK3CD**, PIK3CG	1 / 2
TG-101209	JAK inhibitor	**JAK2**, **JAK3**	preclinical
TGX-221	PI3K inhibitor	PIK3CB, **PIK3CD**	preclinical
TIC10	AKT inhibitor, TRIL modulator	**AKT1**, MAPK1	2
Tofacitinib	JAK inhibitor	**JAK1**, **JAK2**, **JAK3**	launched
Topilutamide	AR antagonist	**AR**	preclinical
Torin-1	mTOR inhibitor	**MTOR**	preclinical
Torin-2	mTOR inhibitor	**MTOR**	preclinical
Tramiprosate	β-amyloid protein neurotoxicity inhibitor	**APP**	3
Triciribine	AKT inhibitor	**AKT1**, AKT2, AKT3	1 / 2

Triflusal	cyclooxygenase inhibitor, platelet aggregation inhibitor, thromboxane synthase inhibitor	**NFKB1**, NOS2, PDE10A, **PTGS1**	launched
Trofinetide	cytokine production inhibitor	**IFNG**, **IL6**, **TNFA**	2
Trometamol	organic amine proton acceptor	AMD1, CANT1, DCN, NEIL1, **VEGFA**	launched
U-0126	MEK inhibitor	**AKT1**, CHEK1, GSK3B, LCK, MAP2K1, MAP2K2, MAP2K7, MAPK1, MAPK11, MAPK12, MAPK14, MAPK8, PRKCA, RAF1, ROCK1, RPS6KB1, SGK1	preclinical
UK-356618	metalloproteinase inhibitor	MMP13, MMP14, MMP2, **MMP3**, **MMP9**	preclinical
Upadacitinib	JAK inhibitor	**JAK1, JAK2, JAK3**	launched
Uprosertib	AKT inhibitor	**AKT1**, AKT2, AKT3	2
Ustekinumab	IL12/IL23 inhibitor	**FSH**, **HCG**, **LH**, **LTA4H**	launched
Vandetanib	EGFR inhibitor, RET tyrosine kinase inhibitor, VEGFR inhibitor	**EGF**R, EPHA1, EPHA10, EPHA2, EPHA3, EPHA4, EPHA5, EPHA6, EPHA7, EPHA8, EPHB1, EPHB2, EPHB3, EPHB4, EPHB6, ERBB2, ERBB3, ERBB4, FLT1, FLT4, KDR, PTK6, RET, SRC, TEK, **VEGFA**	launched
VE-822	ATR kinase inhibitor	ATM, ATR, **MTOR**, PIK3CG	2
Vecabrutinib	Bruton's tyrosine kinase (BTK) inhibitor	**BTK**	1 / 2
Veliflapon	leukotriene synthesis inhibitor	**ALOX5AP**	3
VGX-1027	tumor necrosis factor receptor antagonist	**TLR4**	1
Vercirnon	CC chemokine receptor antagonist	**CCR9**	3
Vicriviroc	CC chemokine receptor antagonist	**CCR5**	3
Voxtalisib	mTOR inhibitor, PI3K inhibitor	**MTOR**, PIK3CA	2
VX-702	p38 MAPK inhibitor	**IL1B**, **IL6**, MAPK11, MAPK12, MAPK14, **TNFA**	2
VX-765	caspase inhibitor	**CASP1**	2
W-54011	anaphylatoxin chemotactic receptor antagonist	**C5AR1**	preclinical
WAY-200070	estrogen receptor agonist	ERBB2, ERBB3, **ERBB4**, ESR2	preclinical
WAY-600	mTOR inhibitor	**MTOR**	preclinical
WHI-P154	JAK inhibitor	**EGFR**, **JAK1**, **JAK2**, **JAK3**	preclinical
Wortmannin	PI3K inhibitor	PI4KA, PI4KB, PIK3CA, **PIK3CD**, PIK3CG, PIK3R1, PLK1, PRKDC	preclinical
WP1066	STAT inhibitor	**STAT3**	1
WP1130	deubiquitinase inhibitor	**JAK2**, UCHL5, USP14, USP9X	preclinical
WYE-125132	mTOR inhibitor	**MTOR**	preclinical
WYE-354	mTOR inhibitor	**MTOR**	preclinical
WYE-687	mTOR inhibitor	**MTOR**	preclinical
XD-14	bromodomain inhibitor	BRD2, BRD3, **BRD4**, BRDT	preclinical
XL019	JAK inhibitor	**JAK1, JAK2, JAK3**	1
XL041	liver X receptor agonist	**LXR**	preclinical
XL147	PI3K inhibitor	PIK3CA, **PIK3CD**, PIK3CG	2
XL388	mTOR inhibitor	**MTOR**	preclinical
Y-320	interleukin inhibitor	**IL17A**	preclinical
YM-90709	IL5 inhibitor	CSF2RB, **IL5**RA	preclinical
Zileuton	leukotriene synthesis inhibitor, lipoxygenase inhibitor	**ALOX5**	launched
ZM-39923	JAK inhibitor	**JAK1**, **JAK3**	preclinical

Zonisamide	sodium channel blocker, T-type calcium channel blocker	CA1, CA10, CA11, CA12, CA13, CA1, CA2, CA3, CA4, CA5A, CA5B, **CA6**, CA7, CA8, CA9, CACNA1G, CACNA1H, CACNA1I, MAOA, MAOB, SCN10A, SCN11A, SCN1A, SCN1B, SCN2A, SCN2B, SCN3A, SCN3B, SCN4A, SCN4B, SCN5A, SCN7A, SCN8A, SCN9A	launched
Zopolrestat	GLO1 inhibitor	AKR1B1, **AKR1B10**	2
ZSTK-474	PI3K inhibitor	PIK3CB, **PIK3CD**, PIK3CG	1 / 2
